# Surface temperature changes indicate disease onset after pulmonary murine corona virus infection, but do not constitute a humane endpoint

**DOI:** 10.1007/s11259-025-10806-9

**Published:** 2025-06-28

**Authors:** Rebecca Nistelberger, Patrizia Gibler, Thomas Filip, Manuel Salzmann, Boris Hartmann, Bruno K. Podesser, Roberto Plasenzotti, Philipp J. Hohensinner, Julia B. Kral-Pointner

**Affiliations:** 1 Core Facility Animal Breeding and Husbandry, Medical University of Vienna, Vienna, Austria; 2 Ludwig Boltzmann Institute for Cardiovascular Research, Vienna, Austria; 3 VetBioModels, University of Veterinary Medicine, Vienna, Austria; 4 Department for Internal Medicine II/Cardiology, Medical University of Vienna, Währinger Gürtel 18–20, Vienna, 1090 Austria; 5 Institute of Veterinary Disease Control, AGES, Mödling, Austria; 6 Center for Biomedical Research and Translational Surgery, Medical University of Vienna, Vienna, Austria

**Keywords:** Surface temperature, Viral infections, Murine coronavirus, Humane endpoint

## Abstract

Mouse models are important contributors for understanding the immune system during infections. Objective parameters help to assess the course of infection and guarantee animal welfare. In this study we analyzed if surface temperature measured via thermal imaging of the dorsal area is a suitable marker to evaluate animal wellbeing during murine coronavirus (MCoV) infection. Infected BALB/c mice displayed severe symptoms whereas C57BL/6 mice were less affected. In BALB/c animals, temperature increased from 27.1 °C to 28.4 °C within 24 h with levels remaining slightly elevated over the observation period. In contrast, a decrease in body weight was consistent through the period with 60% of the animals reaching the previously set termination point of 20% weight loss (*n* = 6). Also, C57BL/6J animals showed a significant temperature increase from 27.1 °C to 28.4 °C within 24 h and a significant weight loss over time with two out of ten reached weight loss end point. However, temperature and weight changes were not related in individual animals. In contrast to temperature values, body weight clearly set a trajectory towards early termination. Taken together, our data indicate that superficial temperature did not serve as a predictive parameter for defining humane end points, but indicate disease onset after pulmonary virus infection.

## Introduction

Monitoring of animal wellbeing is increasingly relevant to avoid unnecessary pain during infection experiments, in which animals often show severe suffering. Therefore, humane endpoints (HEP) are defined, which include the assessment of welfare using animal-based measures such as body weight, clinical signs or coat appearance (Ellis and Katsiadaki 2021). Appropriate behavior with the absence of stress indicators as whisker trimming or bite wounds, absence of injuries as well as absence of lameness, piloerection, and a hunched position indicate that the animals are in good health (Spangenberg and Keeling 2016). Overall, HEP should be used as an alternative to death as an end point ideally incorporating multiple dimensions and clear parameters (Ashall and Millar 2014). Objective HEP parameters, which can be measured include weight loss or temperature. Both of these parameters were described to be suitable in a systematic review in sepsis and stroke animal models (Mei et al. 2019). Additionally, ‘Humane intervention points’ were introduced to describe non-euthanasia responses to enhance animal welfare (Spangenberg and Keeling 2016). Focusing on HEP, temperature was demonstrated to change during infections in mice with core temperature correlating significantly with surface temperature (Mei et al. 2018). Via microchips, core temperature can be measured allowing constant monitoring but implantation implies stress and can cause inflammation (Kort et al. 1998; Warn et al. 2003). To avoid invasive procedures, temperature can also be measured rectally in dorsally fixed animals with the risk of irritation of the rectal mucosa. To prevent tissue damage and animal stress, flat surface temperature probes or non-contact methods were tested (Mei et al. 2018; Vogel et al. 2016; Warn et al. 2003). However, since animals must be restrained, stress can still be induced which might lead to hyperthermia in mice (Blenkus et al. 2022). In contrast, using a thermal imaging camera avoids animal restraining and allows for a larger area of the freely moving animal to be analyzed (van der Vinne et al. 2020).

Measuring body temperature in infectious disease trials can predict early death or unintentional suffering. It is important to include the 3Rs (replacement, reduction and refinement) in an animal study which implicates defining HEPs. Body temperature is a well measurable parameter that might be suitable to define clear cut off values for HEP. During lipopolysaccharide-induced endotoxemia a temperature drop precisely predicted death allowing to set a limit for early euthanisation to prevent unnecessary distress (Mei et al. 2018). The aim of our study was to determine if measuring the temperature via infrared thermography during a mouse coronavirus infection (MCoV) could serve as a parameter of animal wellbeing and can be added as an ‘objective’ criteria to HEPs in viral infection trials. More specifically, we wanted to determine if mouse surface temperature is a predictive marker for worsening of disease progression preceding weight loss thereby increasing animal well-being through earlier end point definitions.

## Materials and methods

### Animals

Mice (Janvier, France), were housed in IVC cages (Tecniplast, Germany), provided with commercially available food (Altromin, Germany) with aspen wood as bedding (Las Vendi, Germany). Animals were housed in a climate-controlled room at 22.5–23 °C with 55% (± 10%) humidity controlled continuously via dataloggers (Testo GmbH, Austria), and 12-h day/12-h night light cycle. Animals had access to food and autoclaved water ad libitum. Six BALB/c and 20 C57BL/6J mice (equal sex distribution, 8–10 weeks age) were used. Primary HEP was weight loss > 20%. Mice were infected with the murine coronavirus (MCoV), previously known as mouse hepatitis virus (MHV) using the sub-strain A59. Infection of animals was performed as published previously (Salzmann et al. 2022). In short, mice were inoculated with 10^6^ TCID50 MCoV intranasally under general anesthesia using 0.5 mg/kg medetomidine (Domitor^®^, Orion Pharma) and 5 mg/kg midazolam (Accord) administered intraperitoneally. Antagonization, if required, was performed with 2.5 mg/kg atipamezole (Antisedan^®^, Orion Pharma) and 0.5 mg/kg flumazenil (Pharmaselect) subcutaneously.

### Temperature measurements

**Fig. 1:**
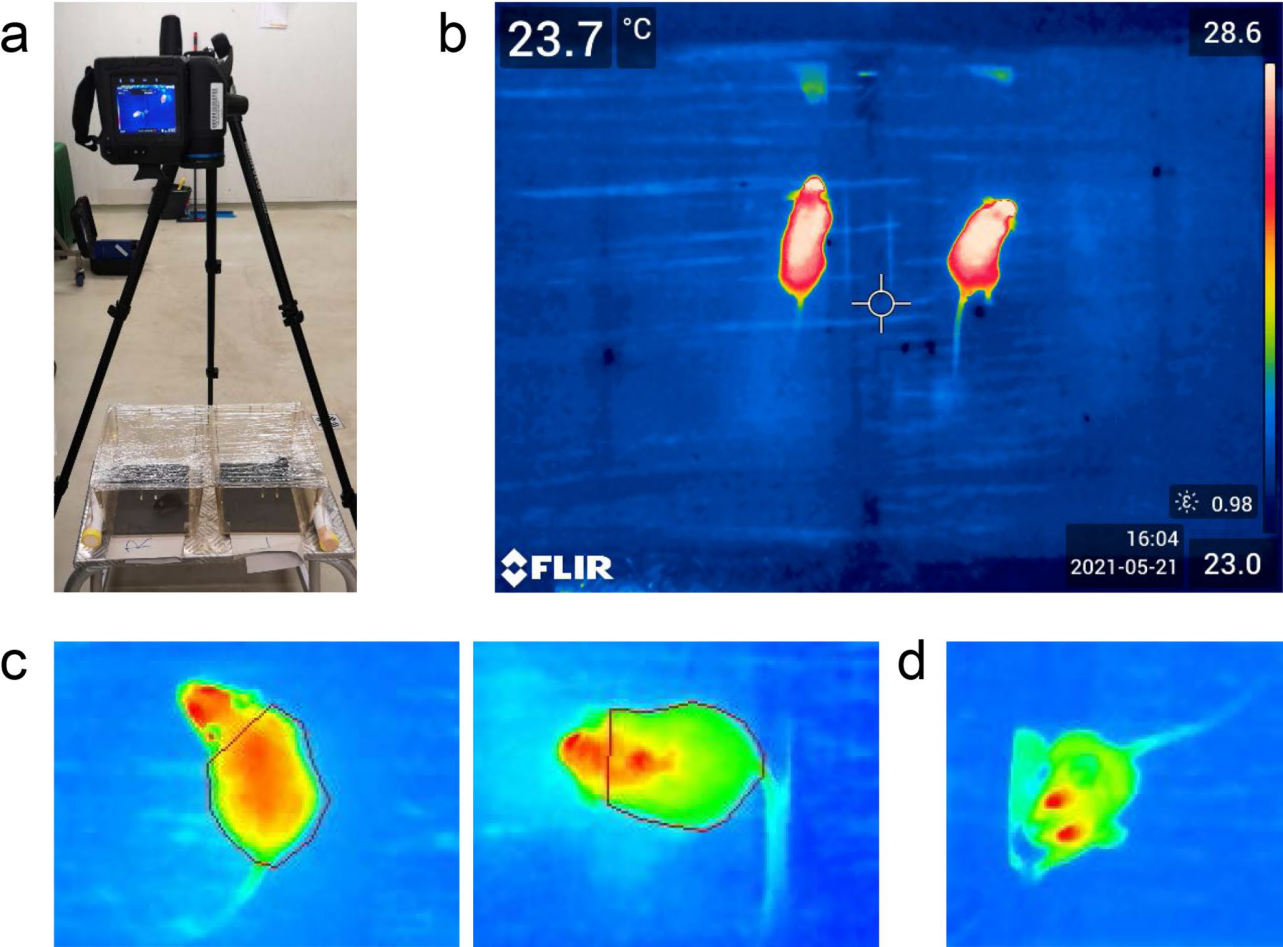
Technical setup of surface temperature measurement (**a**) Example of surface temperature measurement via thermal imaging of the dorsal area and (**b**) camera setting. (**c**) Representative images depicting the region of interest of a selected C57BL/6J (left) and a BALB/c (right) mouse. (**d**) Picture of a mouse that cannot be included due to animal positioning

Superficial temperature of the animals was detected using an infrared camera (FLIR T865, FLIR wide angle lens f = 10 mm, Flir Systems, OR, USA) with a 5-minutes warm up period. Previously, Verduzco-Mendoza et al. suggested several regions for temperature measurement (Verduzco-Mendoza et al. 2021). We expanded the interscapular window to the whole dorsal area to obtain a more unified observation area. Of note, the brown adipose tissue area is visible as an area of high temperature in our experimental setting. Measurements were taken at two time points, the morning measurement starting at 10:00 am and the afternoon measurement starting at 2:30 pm. For measurements, animals were transferred into two empty and clean type 2 cages (Tecniplast, Germany), covered with a thin, transparent foil for a total of 2 min. The foil was used to retain animals within the cage and reduced the measured temperature by 4.6%±1 as determined in a preliminary experiment using 10 uninfected animals. The camera was mounted 70 cm above the cage floor to allow a full view of the cage. During measurement (2 min period, 12 pictures, every 10 s) a single mouse was placed in a single cage allowing for the measurements of two animals next to each other at the same time (overview of setup in Fig. 1a and b). Animals were transferred via tunnel handling without direct touch to avoid temperature transfer or stress. For temperature quantification, the area of interest was selected manually for each picture using the program provided by the camera supplier (FLIR). Only pictures where a clear outline of the animal was visible were used to monitor temperature (Fig. 1c). An example of the defined region of interest (ROI) is given in Fig. 1c demonstrating the start of the selected ROI behind the head including the brown adipose tissue section in the analysis with the average temperature of the area used for analysis. The best 5 images from one measurement setting per mouse (focus and sharpness) were taken for analyses. The picture analysis was performed in a blinded manner by a research team member. Figure 1d gives an example of an image that was excluded from the analysis.

### Study design

This study was designed with behavioral changes and weight loss as the major factors of HEP. Temperature was used as an additional test parameter. Temperature changes and weight curves were analyzed over all animals within one experiment to determine if the average weight or temperature would significantly change during the experiment. Data were compared as indicated in the figures using GraphPad Prism8 with *p* < 0.05 considered as statistically significant (GraphPad Software, CA, USA). Data are given as average ± standard deviation if not indicated otherwise.

## Results

**Fig. 2:**
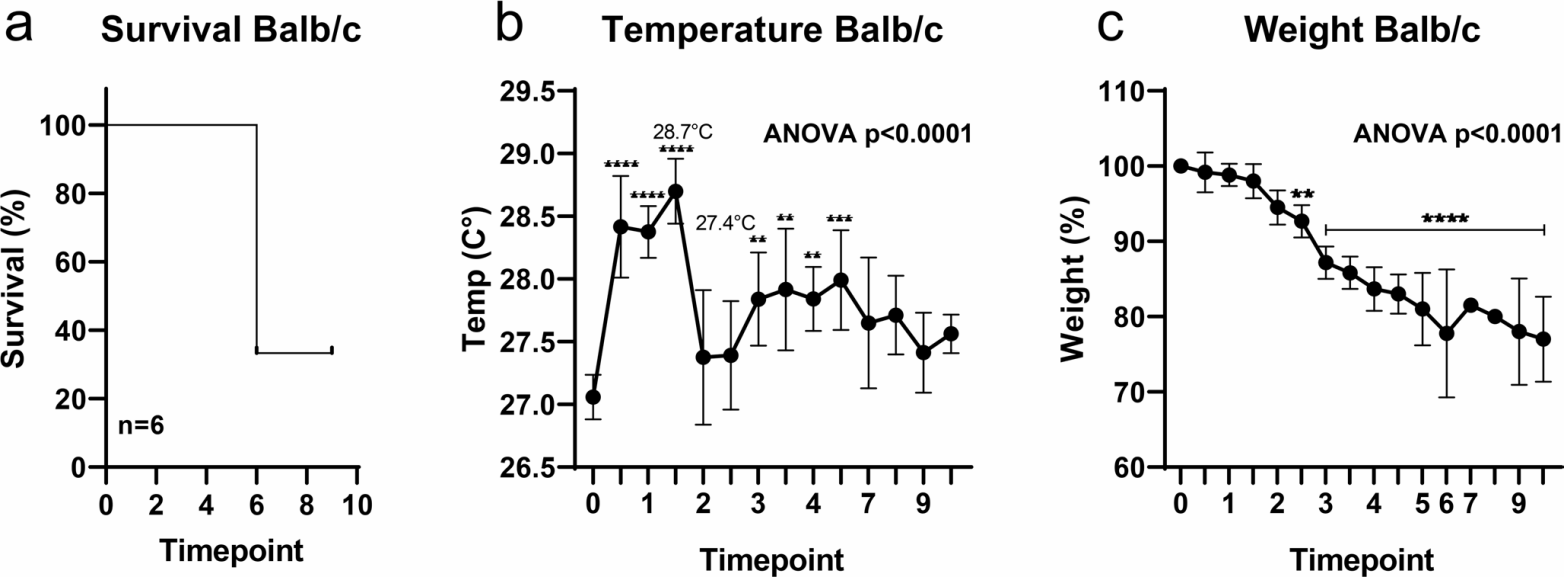
Temperature measurements in BALB/c mice during Murine Corona Virus infection (MCoV). BALB/c mice were infected with MCoV at day 0 and monitored over 10 days. Temperature and weight were measured twice a day. During MCoV infection, (**a**) more than 50% of the animals were lost during the observation period. (**b**) Surface temperature increased initially but was not statistically significant at later time points. (**c**) Weight loss started at day 2 and continued over the whole observation period. One-way ANOVA with Dunnett post hoc test was used to determine significance. *n* = 6. *p* < 0.05 is considered significant. **p < 0.01,***p < 0.005, ****p < 0.001

Weight control is a standard parameter in determination of HEP and also in determining the immune response of animals towards a virus infection. Depending on the virus, mouse strains behave differently. To evaluate if temperature might be added as another predictive parameter for HEP we analyzed temperature changes in BALB/c and C57BL/6J after MCoV infection. Challenging BALB/c animals with 10^6^ TCID_50_ MCoV led to a lethality rate over 50% (Fig. 2a). However, this was not due to spontaneous death but was required according to HEP as weight loss reached the limit of 20%. Temperature demonstrated an increase of up to 1.65 °C for the initial 24 h afterwards dropping towards baseline with a slightly elevated temperature over the observation period (Fig. 2b). During the observation period we found a significant and constant weight loss starting at day 2 and continuing over the remaining days (Fig. 2c). Temperature remained similar for all animals regardless of weight variation. Temperature of animals at the time of euthanisation was on average 27.7 °C in contrast to 27.4 °C for the remaining animals (*p* = 0.62).

**Fig. 3:**
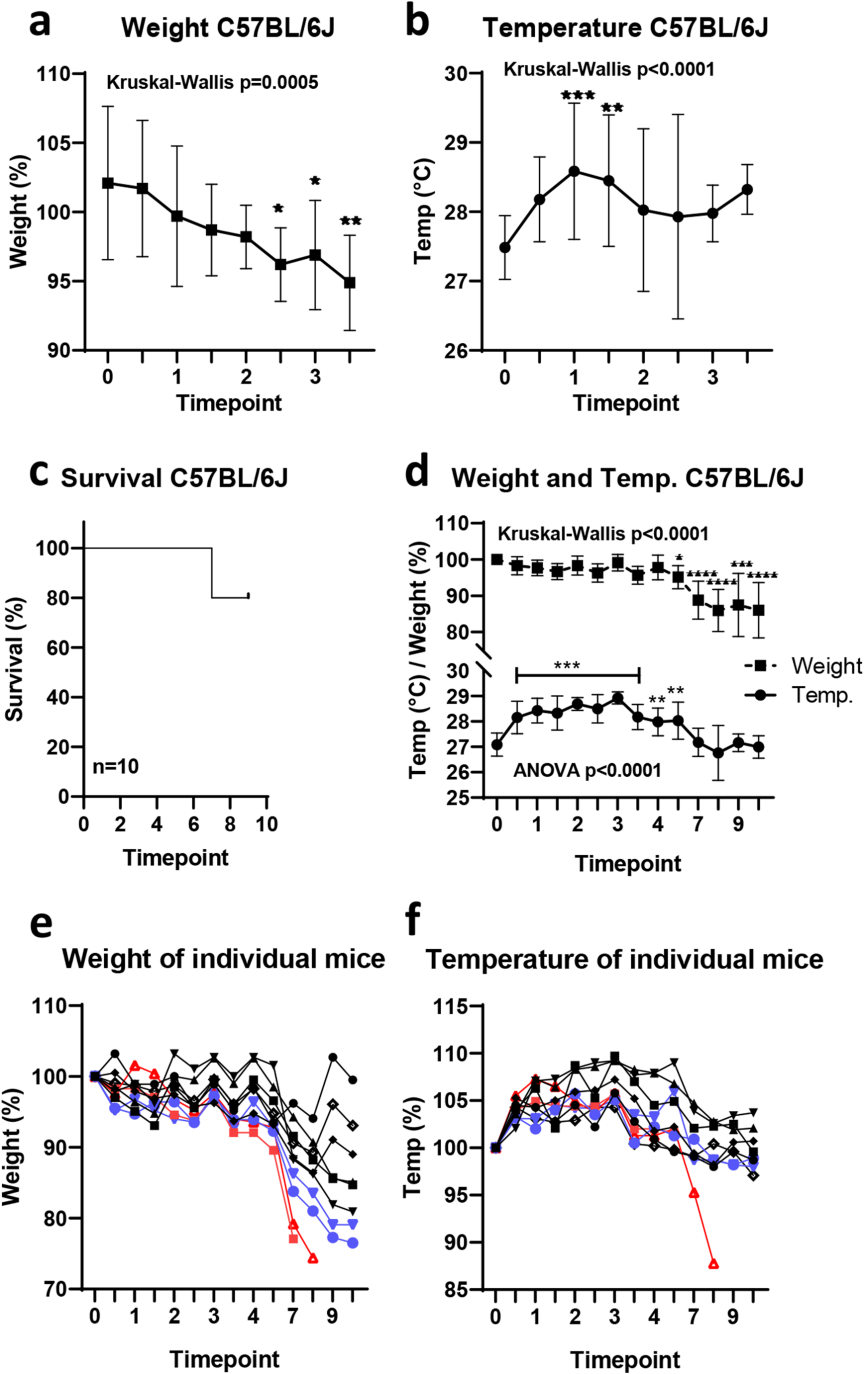
Temperature measurement in C57BL/6J mice during Murine Corona Virus infection (MCoV). C57BL/6J mice were infected with MCoV at day 0 and monitored over 3 (**a**-**b**) or 9 (**c**-**f**) days. During the short-term experiment, we observed minimal changes in weight (**a**) and a slight increase in body temperature (Temp.) (**b**). During a longer observation period, 80% of the animals survived the virus infection (**c**). We found weight loss towards the later time points with elevated temperatures for the first day (**d**). (**e**-**f**) Percentage values of individual mice of body weight (**e**) and surface temperature (**f**). Red symbols indicate mice, which reached the humane endpoint (HEP) criteria of 20% body weight loss before the end of the experiment and blue symbols indicate mice, which reached 20% weight loss on the harvest day. We observed that body weight in contrast to the surface temperature already declined before the threshold of HEP was met. One-way ANOVA with Dunnett post hoc test or Kruskal-Wallis with Dunn’s post hoc test was used to determine significance. *n* = 10. *p* < 0.05 is considered significant. *p < 0.05, **p < 0.01,***p < 0.005, ****p < 0.001

In contrast to BLAB/c, C57BL/6J mice are more robust towards a MCoV infection. During a short-term observation of the animals for three days, we observed only minimal weight changes of 5% (Fig. 3a). Similarly, superficial temperature was only moderately affected by the infection (Fig. 3b).

When observing C57BL/6J over ten days, two out of ten mice were sacrificed due to the HEP criteria of 20% weight loss (Fig. 3c) and two further mice reached this threshold on the harvest day (Fig. 3e). Overall, mice lost gradually weight with significant weight loss over time especially towards the end of the observation period (Fig. 3d). Temperature was significantly elevated for the first four days but returned to normal at later time points while body weight declined further and reached statistical significance (Fig. 3d). Looking at individual mice weight measurements (Fig. 3e) helped to predict the wellbeing of a mouse, as a gradual weight loss was observed in mice reaching the HEP criteria of 20% weight loss. In contrast, temperature levels (Fig. 3f) of these mice were aligned similar with surviving mice, not hinting towards a survival risk. Only one out of four mice had a drop in temperature levels at the day the HEP was reached and clinical signs of suffering occurred. Thus, our data showed that temperature did not constitute a predictive marker to broaden the spectrum of HEPs. However, a temperature increase after 24 h was quite consistent over the three cohorts may reflecting the onset of viral infection. Thus, superficial temperature measurement might be used to monitor the onset of infections.

## Discussion

In conclusion, superficial body temperature was slightly, but significant increased in both mouse strains one day after virus infection. For both mouse strains, weight loss was continuing over the observation period with temperature levels returning to baseline values at later stages of the observation period. Interestingly, in a Roborovski hamster model it was reported that also a SARS-CoV-2 coronavirus infection induced a fever reaction in a laboratory setting (Zhai et al. 2021). Fever in general refers to an acute phase response that confers a survival benefit on the body, raising core body temperature during infection or systemic inflammation (Mota-Rojas et al. 2021). For mice, fever induction was reported in an influenza virus model with temperature increase directly related to IL-1α production (Kurokawa et al. 1996). Interestingly, in our infection model weight loss continued whereas temperature measurements returned to the starting temperature. Thus, superficial temperature measurement could be a tool to monitor the course of infection, especially the onset, and could thereby function as an infection control.

Nonetheless, in both settings, temperature was not predictive for weight loss in a MCoV model. Talbot et al. suggested to add parameters to weight loss for a larger evaluation spectrum of distress. (Talbot et al. 2020). However, our data do not indicate that surface temperature is a valid parameter during MCoV infection in mice to predict animal distress. Although measuring the superficial temperature of mice would be an objective method with low stress potential, body weight and evaluation of clinical signs demonstrated a clearer view on the well-being of the animal.

BALB/c animals were already published to be affected strongly by MCoV whereas C57BL/6J animals can cope well with the infection (Korner et al. 2020). Using these two extreme mouse strains did not reveal a strain-dependent temperature effect with slightly elevated surface temperatures after infection in both mouse strains but this temperature elevation did not predict weight loss.

We conclude that superficial temperature measurements neither serve as a substitute for weight measurements nor show a meaningful correlation with them and thus, does not constitute an appropriate HEP criterion. However, superficial body temperature of mice is affected by MCoV infections may indicating disease onset after intranasal MCoV infection and may serve as an infection control.

## Limitations

A significant limitation of the presented study is the relatively low sample size, which may impact the statistical power and generalizability of the findings. However, since our findings discourage the use surface body temperature as predictor for humane endpoint, the emphasis of future studies may be placed on alternative clinical indicators and their objective assessment including larger and more diverse cohorts.

## Data Availability

Full datasets can be obtained from the corresponding author upon reasonable request.
